# (6*R*)-2-*tert*-Butyl-6-[(4*R*,5*S*)-3-isopropyl-4-methyl-5-phenyl­oxazolidin-2-yl]phenol

**DOI:** 10.1107/S1600536810009591

**Published:** 2010-03-24

**Authors:** Takaoki Koyanagi, Kate L. Edler, Raleigh W. Parrott, Shawn R. Hitchcock, Gregory M. Ferrence

**Affiliations:** a CB 4160, Department of Chemistry, Illinois State University, Normal, IL 61790, USA

## Abstract

In the title compound, C_23_H_31_NO_2_, the lone pair on the nitro­gen atom is oriented to facilitate intra­molecular hydrogen bonding with the hydr­oxy group residing on the phenyl substituent. The five-membered ring adopts an envelope confornmation with the O atom at the flap. The absolute stereochemistry was verified by measurement of optical activity using a digital polarimeter.

## Related literature

For related structures and background to the use of chiral oxazolidines in asymmetric synthesis, see: Agami & Couty (2004 ▶); Anderson *et al.* (2010 ▶); Campbell *et al.* (2010 ▶); Ge *et al.* (2003 ▶); Hitchcock *et al.* (2004 ▶); Nakano *et al.* (2001 ▶); Parrott *et al.* (2008 ▶); Parrott & Hitchcock (2007 ▶). For geometry checks using *Mogul*, see: Bruno *et al.* (2004 ▶). For ring puckering analysis, see: Boeyens (1978 ▶); Cremer & Pople (1975 ▶); Spek (2009 ▶). For a description of the *Jmol* toolkit for the preparation of enhanced figures, see: McMahon & Hanson (2008 ▶).
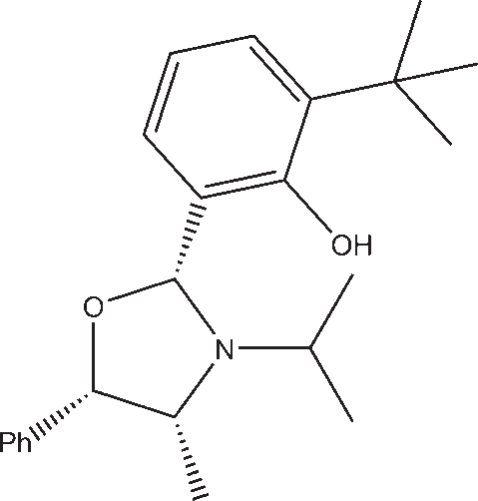


## Experimental

### 

#### Crystal data

C_23_H_31_NO_2_
*M* *_r_* = 353.49
Monoclinic, 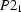

*a* = 9.5077 (6) Å
*b* = 7.3257 (5) Å
*c* = 14.983 (1) Å
β = 101.615 (1)°
*V* = 1022.20 (12) Å^3^
*Z* = 2
Mo *K*α radiation
μ = 0.07 mm^−1^
*T* = 140 K
0.53 × 0.41 × 0.39 mm

#### Data collection

Bruker SMART APEX CCD diffractometer
Absorption correction: multi-scan (*SADABS*; Bruker, 2008 ▶) *T*_min_ = 0.823, *T*_max_ = 0.972
9840 measured reflections
2537 independent reflections
2445 reflections with *I* > 2σ(*I*)
*R*_int_ = 0.019

#### Refinement

*R*[*F* ^2^ > 2σ(*F* ^2^)] = 0.03
*wR*(*F* ^2^) = 0.082
*S* = 1.03
2537 reflections
239 parameters
1 restraint
H atoms treated by a mixture of independent and constrained refinement
Δρ_max_ = 0.22 e Å^−3^
Δρ_min_ = −0.15 e Å^−3^

### 

Data collection: *APEX2* (Bruker, 2008 ▶); cell refinement: *APEX2* and *SAINT* (Bruker, 2008 ▶); data reduction: *SAINT*; program(s) used to solve structure: *SIR2004* (Burla *et al.*, 2005 ▶); program(s) used to refine structure: *SHELXL97* (Sheldrick, 2008 ▶); molecular graphics: *ORTEP-3* for Windows (Farrugia, 1997 ▶) and *Mercury* (Macrae *et al.*, 2008 ▶); software used to prepare material for publication: *WinGX* (Farrugia, 1999 ▶) and *publCIF* (McMahon & Westrip, 2008 ▶).

## Supplementary Material

Crystal structure: contains datablocks global, I. DOI: 10.1107/S1600536810009591/zl2268sup1.cif

Structure factors: contains datablocks I. DOI: 10.1107/S1600536810009591/zl2268Isup2.hkl

Additional supplementary materials:  crystallographic information; 3D view; checkCIF report

**Fig. 2.**
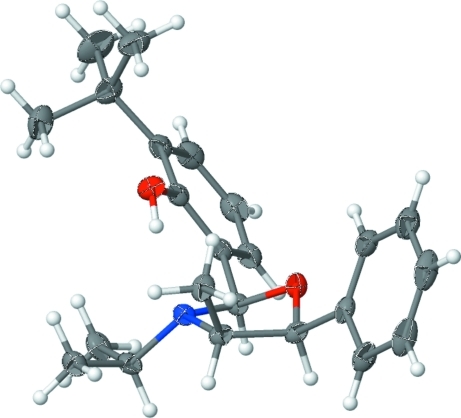
The enhanced Jmol figure of the title compound. The intramolecular H-bonding is highlighted as one of the radiobuttons. This is the first in a series of three Jmol figures intended to illustrate some versatility of the program. See also: Campbell et al. (2010); Anderson et al. (2010). In this Jmol, all interactive features are defined by using the graphical interface. Some script artifacts occur and can only be remedied by hand-editing the scripts.

Enhanced figure: interactive version of Fig. 2

## Figures and Tables

**Table 1:** Hydrogen-bond geometry (Å, °)

*D*—H⋯*A*	*D*—H	H⋯*A*	*D*⋯*A*	*D*—H⋯*A*
O22—H22⋯N3	0.85 (3)	1.84 (2)	2.6280 (16)	154 (2)

**Fig. 1.**
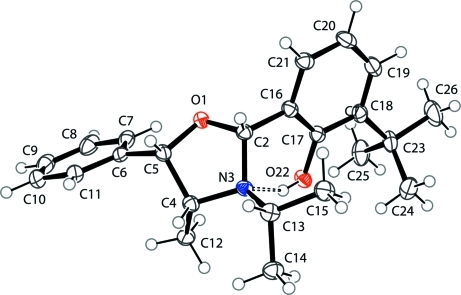
The molecular structure of the title compound with the atomic numbering scheme and intramolecular H-bonding. Displacement ellipsoids are drawn at the 50% probability level.

## supplementary crystallographic information

### Comment

Chiral oxazolidines are useful templates for conducting asymmetric syntheses (Agami & Couty, 2004). In order to explore the utility of these compounds in the catalytic asymmetric addition of diethylzinc to aldehydes, we prepared a series of oxazolidines from (1*R*,2S)-ephedrine (Parrott & Hitchcock, 2007), (1*R*,2S)-norephedrine (Parrott *et al.*, 2008), and (1*S*,2*R*)-norephedrine (this paper). In the course of synthesizing these oxazolidines, we were able to obtain crystals suitable for X-ray crystallographic analysis.

Additional oxazolidine systems have been reported and studied (Parrott *et al.*, 2008), where the phenyl substituent has a hydrogen atom alpha to the hydroxyl group. The torsion angles of the title compound mostly agree with this unsubstituted oxazolidine. Two minor differences arise that may be due to the sterically enhanced phenyl group. The oxazolidine compound previously reported (Parrott *et al.*, 2008) contained a C21—C2—N3—C4 torsion angle being equal to 156.8 (2)°, while the corresponding title compound torsion angle of C4—N3—C2—C16 is equal to 147.10 (12)°. The second torsion angle of the reported oxazolidine (Parrott *et al.*, 2008), C21—C2—N3—C31, is equal to -72.8 (3)°, whereas the title oxazolidine has a torsion angle equal to -87.85 (14)° at C13—N3—C2—C16. The molecular structure shown in Fig. 1 has one molecule in the asymmetric unit. A *Mogul* geometry check (Bruno *et al.*, 2004) shows the only unusual bond length or bond angle to be the C5—O1—C2 angle with a value of 103.95 (11)° against a mean of 107.8°.

Ring puckering analysis using *PLATON* (Spek, 2009; Cremer & Pople, 1975; Boeyens, 1978) indicates Φ = 1.63 (17)° for the O1—C2—N3—C4—C5 ring, which is consistent with a formal conformational assignment close to an idealized ^1^E envelope with O1 being the flap apex. The crystal structure suggests that the isopropyl group on N3 has an *anti*-relationship with the substituents on C2, C4, and C5 due to the intramolecular H-bonding interaction between N3 and the hydroxyl group. The donor to acceptor atom distance (2.6278 (16) Å) between O22—N3 is large enough to only support a weak H-bonding interaction. This interaction is further illustrated in the Jmol enhanced figure (Fig. 2).

About the Jmol enhanced figure:

The procedure for recreating the Jmol figure is provided in the hopes that readers will find it useful for creating their own. We are reporting three related structures containing Jmol enhanced figures, one in this paper and the other two in other papers in this *Journal* (Campbell *et al.*, 2010; Anderson *et al.*, 2010). The Jmol enhanced figures were created to illustrate a range of author convenience versus end user experience, ranging from a purely GUI driven experience for the author resulting in a less functional figure for the end user to a more sophisticated use of the Jmol scripting by the author resulting in a more polished and versatile figure for the end user. The buttons, check boxes and radio buttons in the three examples visually appear to be identical; however, the underlying code they execute results in significantly different overall responses by the Jmol visualizer.

By strictly authoring with the Jmol toolkit GUI, without text editing any code, generation of the figure is relatively quick and easy. However, doing so results in a final figure which has some significant limitations. In particular, when the end user manipulates the figure by, for example, a rotation, subsequent clicking of a radiobutton will result in the figure resetting to appear exactly as it appeared when the author saved the script. This includes all settings such as orientation and any other highlighting. This is the scenario illustrated by the Jmol enhanced figure associated with this Acta E article. The enhanced figure options were intentionally selected without any alteration of the structure's orientation, so that as long as the user does not move or rotate the structure, the molecule's orientation appears static.

The Jmol options were created as follows:

Labels were added to atoms by navigating to the "label" sub-tab under the "select/label" tab and by checking the button "atom name" before turning the labels "on". The script was imported into a checkbox by navigating to the "checkbox" sub-tab under the "script" tab, and by clicking "import view".

The thermal displacement coloring was achieved by navigating to the "model" tab and by selecting "atomic displacement" next to the "colour" heading.

The color of particular atoms was changed by first selecting them. The atoms were selected by navigating to the "select/label" tab, turning the "highlight selection" on, and picking "within area" under "selection mode". The color of the atoms was changed by navigating to the "atoms" sub-tab and picking a color from the drop down box next to the "colour" heading.

The various atom styles were selected by navigating to the "model" tab and by selecting the atom style of choice next to the "overall style" heading.

The hydrogen bond was displayed by navigating to the "measurements" sub-tab under the "select/label" tab. The "distance" option next to the "mode" heading was then selected, followed by the hydrogen and acceptor atoms.

### Experimental

The title compound was synthesized in two steps. Optical activities were measured at 589 nm using a digital polarimeter as discribed for similar compounds in Parrott & Hitchcock (2007). The synthesis included reagents and solvents of reagent grade, which were used without further purification.

In the first step, to a flame dried, nitrogen purged flask was added (1*S*,2*R*)-norephedrine (10.1 g, 66.8 mmol), ethanol (100 ml), and acetone (7.4 ml, 100 mmol). The mixture was allowed to stir at room temperature for 24 hours. At that time the solution was cooled to 273 K and sodium borohydride (5.07 g, 134 mmol) was added and the mixture allowed to stir for 2 hours. The ethanol was removed under reduced pressure and the reaction quenched with sodium hydroxide (1M, 100 ml). The product was extracted with ethyl acetate (100 ml × 2), washed with brine, dried with magnesium sulfate, gravity filtered, and concentrated under reduced pressure. The amino alcohol was purified via recrystallization with hexanes: ethyl acetate (2:1) to afford (1-*S*,2*R*)-2-isopropylamino-1-phenyl-1-propanol as a white solid in 65% yield. [α]_D_^25^ = 10.3 (*c* 1.28, CHCl_3_). Mp = 372-374 K. ^1^H NMR: δ 0.80 (d, *J* = 6.6 Hz, 3H), 1.10 (overlapping doublets, *J* = 6.6 Hz, 6H), 2.97 (m, 1H), 3.05 (dq, *J* = 3.9, 6.3 Hz, 1H), 4.69 (d, *J* = 3.9 Hz, 1H), 7.23-7.35 (m, 5H). ^13^C {^1^H} NMR (CDCl_3_): d 15.0, 23.4, 23.5, 45.5, 55.1, 73.5, 126.1, 126.8, 127.9, 141.6. IR (CHCl_3_): 3431, 1124, 1082, 743, 705. ESI-HRMS Calcd for C_12_H_20_N_1_O_1_ (M^+^ + H): 194.1545. Found: 194.1549.

In the second step, to a flame dried, nitrogen purged flask was added (1-*S*,2*R*)-2-isopropylamino-1-phenyl-1-propanol (2.05 g, 10.6 mmol), methanol (45 ml), 2-hydroxy-3-*tert*butylbenzaldehyde (1.89 g, 10.6 mmol), and sodium sulfate (7.50 g, 53.2 mmol). The mixture was stirred under reflux for 17 h then filtered through Celite. Excess solvent was removed under reduced pressure and the product was recrystallized with ethyl ether and hexanes (1:2) to afford the title compound as white crystals in 6% yield. [α]_D_^25^ = 5.0 (*c* 0.10, CHCl_3_). Mp = 409-410 K. ^1^H NMR (CDCl_3_): δ 0.90 (d, *J* = 6.6 Hz, 3H), 1.14 (d, *J* = 6.6 Hz, 3H), 1.20 (d, *J* = 6.3 Hz, 3H), 1.44 (s, 9H), 3.11 (septet, *J* = 6.6 Hz, 1H). 3.56 (pentet, *J* = 6.6 Hz, 1H), 5.09 (d, *J* = 7.0 Hz, 1H), 5.36 (s, 1H), 6.76 (t, *J* = 7.8 Hz, 1H), 7.05 (dd, *J* = 1.6,7.4 Hz, 1H), 7.24-7.35 (m, 6H), 12.40 (br s, 1H). ^13^C {^1^H} NMR (CDCl_3_): δ 18.7 19.2, 21.3, 29.4, 34.8, 50.3, 57.5, 81.2, 95.8, 117.8, 120.6, 126.6, 127.4, 127.6, 128.1, 128.3, 137.0, 137.1, 158.1. IR (Nujol mull): 3442, 1605, 1592, 1174, 752, 712, 701 cm^-1^. ESI-HRMS Calcd for C_23_H_32_N_1_O_2_ (M^+^ + H): 354.2433. Found 354.2445.

Single crystals of the title compound were grown by vapor diffusion of hexane into a methylene chloride solution of the title compound.

### Refinement

All non-H atoms were refined anisotropically without disorder. The absolute configuration of the title compound is based on the known stereochemistry of the commercially obtained optically pure norephedrine from which it was prepared and optical activity was measured as discribed for similar compounds in Parrott & Hitchcock (2007). All H atoms were initially identified through difference Fourier syntheses then, except for the O–H hydrogen atom, removed and included in the refinement in the riding-model approximation (C–H = 0.95, 0.98, and 1.00 Å for Ar–H, CH_3_ and CH; Uiso(H) = 1.2Ueq(C) except for methyl groups, where Uiso(H) = 1.5Ueq(C)). The OH H atom was freely refined isotropically. In the absence of significant anomalous scattering effects, Friedel pairs were merged.

### Crystal data

**Table:**

C_23_H_31_NO_2_	*F*(000) = 384
*M**_r_* = 353.49	*D*_x_ = 1.148 Mg m^−^^3^
Monoclinic, *P*2_1_	Mo *K*α radiation, λ = 0.71073 Å
Hall symbol: P 2yb	Cell parameters from 8065 reflections
*a* = 9.5077 (6) Å	θ = 2.3–31.2°
*b* = 7.3257 (5) Å	µ = 0.07 mm^−^^1^
*c* = 14.983 (1) Å	*T* = 140 K
β = 101.615 (1)°	Block, colourless
*V* = 1022.20 (12) Å^3^	0.53 × 0.41 × 0.39 mm
*Z* = 2	

### Data collection

**Table:**

Bruker SMART APEX CCD diffractometer	2445 reflections with *I* > 2σ(*I*)
ω scans	*R*_int_ = 0.019
Absorption correction: multi-scan (*SADABS*; Bruker, 2008)	θ_max_ = 27.5°, θ_min_ = 1.4°
*T*_min_ = 0.823, *T*_max_ = 0.972	*h* = −12→12
9840 measured reflections	*k* = −9→9
2537 independent reflections	*l* = −19→19

### Refinement

**Table:**

Refinement on *F*^2^	1 restraint
Least-squares matrix: full	H atoms treated by a mixture of independent and constrained refinement
*R*[*F*^2^ > 2σ(*F*^2^)] = 0.03	*w* = 1/[σ^2^(*F*_o_^2^) + (0.0516*P*)^2^ + 0.1569*P*] where *P* = (*F*_o_^2^ + 2*F*_c_^2^)/3
*wR*(*F*^2^) = 0.082	(Δ/σ)_max_ < 0.001
*S* = 1.03	Δρ_max_ = 0.22 e Å^−^^3^
2537 reflections	Δρ_min_ = −0.15 e Å^−^^3^
239 parameters	

### Special details

**Table:**

Geometry. All esds (except the esd in the dihedral angle between two l.s. planes) are estimated using the full covariance matrix. The cell esds are taken into account individually in the estimation of esds in distances, angles and torsion angles; correlations between esds in cell parameters are only used when they are defined by crystal symmetry. An approximate (isotropic) treatment of cell esds is used for estimating esds involving l.s. planes.

### Fractional atomic coordinates and isotropic or equivalent isotropic displacement parameters (Å^2^)

**Table:**

	*x*	*y*	*z*	*U*_iso_*/*U*_eq_	
O1	0.09280 (10)	0.05027 (15)	0.41402 (6)	0.0211 (2)	
O22	0.31418 (10)	0.10290 (16)	0.25925 (7)	0.0232 (2)	
N3	0.18897 (12)	0.30937 (18)	0.36374 (8)	0.0188 (2)	
C16	0.06048 (15)	0.07792 (19)	0.25140 (9)	0.0202 (3)	
C17	0.18240 (15)	0.0483 (2)	0.21331 (9)	0.0199 (3)	
C2	0.06770 (14)	0.1778 (2)	0.34047 (9)	0.0192 (3)	
H2	−0.0251	0.2429	0.3394	0.023*	
C18	0.16944 (16)	−0.0342 (2)	0.12667 (9)	0.0232 (3)	
C7	0.25468 (16)	−0.1201 (2)	0.56806 (11)	0.0261 (3)	
H7	0.2477	−0.1741	0.5097	0.031*	
C5	0.13056 (14)	0.1627 (2)	0.49354 (9)	0.0205 (3)	
H5	0.0424	0.2249	0.5052	0.025*	
C12	0.39146 (14)	0.2626 (2)	0.49450 (10)	0.0241 (3)	
H12A	0.4486	0.3598	0.4743	0.036*	
H12B	0.4116	0.1466	0.467	0.036*	
H12C	0.4165	0.2524	0.561	0.036*	
C11	0.20485 (16)	0.1286 (3)	0.66191 (10)	0.0287 (3)	
H11	0.1633	0.2448	0.6677	0.034*	
C19	0.03264 (18)	−0.0911 (2)	0.08297 (10)	0.0277 (3)	
H19	0.0214	−0.1482	0.025	0.033*	
C8	0.32400 (16)	−0.2135 (3)	0.64589 (11)	0.0336 (4)	
H8	0.3641	−0.3308	0.6404	0.04*	
C21	−0.07376 (15)	0.0199 (2)	0.20467 (10)	0.0240 (3)	
H21	−0.1562	0.0401	0.2301	0.029*	
C6	0.19613 (14)	0.0511 (2)	0.57569 (9)	0.0220 (3)	
C13	0.15692 (15)	0.4965 (2)	0.32618 (10)	0.0227 (3)	
H13	0.0848	0.5542	0.3575	0.027*	
C14	0.29374 (16)	0.6112 (2)	0.34524 (11)	0.0264 (3)	
H14A	0.3331	0.6128	0.4109	0.04*	
H14B	0.2714	0.7363	0.3237	0.04*	
H14C	0.3645	0.5583	0.3133	0.04*	
C9	0.33429 (17)	−0.1348 (3)	0.73133 (11)	0.0382 (5)	
H9	0.3823	−0.1975	0.7842	0.046*	
C4	0.23252 (14)	0.3075 (2)	0.46524 (9)	0.0199 (3)	
H4	0.2128	0.4296	0.49	0.024*	
C23	0.30071 (18)	−0.0529 (3)	0.08149 (10)	0.0293 (4)	
C15	0.09567 (18)	0.4936 (2)	0.22390 (10)	0.0293 (3)	
H15A	0.0077	0.4202	0.2117	0.044*	
H15B	0.1663	0.4401	0.1921	0.044*	
H15C	0.0737	0.6185	0.2022	0.044*	
C20	−0.08768 (17)	−0.0671 (2)	0.12135 (11)	0.0280 (3)	
H20	−0.1789	−0.1101	0.0905	0.034*	
C25	0.41693 (19)	−0.1736 (3)	0.13943 (11)	0.0374 (4)	
H25A	0.4993	−0.1833	0.1096	0.056*	
H25B	0.3776	−0.2955	0.1458	0.056*	
H25C	0.4478	−0.1187	0.1999	0.056*	
C10	0.27441 (18)	0.0356 (3)	0.73926 (11)	0.0364 (4)	
H10	0.2809	0.089	0.7977	0.044*	
C24	0.3624 (2)	0.1375 (3)	0.06962 (12)	0.0380 (4)	
H24A	0.4461	0.1258	0.0411	0.057*	
H24B	0.3913	0.1958	0.1294	0.057*	
H24C	0.2891	0.2122	0.0308	0.057*	
C26	0.2602 (2)	−0.1396 (4)	−0.01343 (12)	0.0464 (5)	
H26A	0.3459	−0.1496	−0.04	0.07*	
H26B	0.1886	−0.0632	−0.0526	0.07*	
H26C	0.2201	−0.2615	−0.0083	0.07*	
H22	0.301 (2)	0.178 (4)	0.3002 (15)	0.039 (6)*	

### Atomic displacement parameters (Å^2^)

**Table:**

	*U*^11^	*U*^22^	*U*^33^	*U*^12^	*U*^13^	*U*^23^
O1	0.0221 (5)	0.0231 (5)	0.0171 (4)	−0.0026 (4)	0.0016 (3)	0.0013 (4)
O22	0.0192 (5)	0.0296 (6)	0.0200 (5)	0.0013 (4)	0.0017 (4)	−0.0037 (5)
N3	0.0171 (5)	0.0183 (6)	0.0196 (5)	0.0004 (5)	0.0007 (4)	0.0005 (5)
C16	0.0218 (6)	0.0182 (7)	0.0189 (6)	0.0006 (5)	0.0005 (5)	0.0016 (5)
C17	0.0210 (6)	0.0186 (6)	0.0186 (6)	0.0013 (5)	0.0002 (5)	0.0027 (5)
C2	0.0163 (6)	0.0207 (7)	0.0199 (6)	0.0001 (5)	0.0016 (5)	0.0018 (5)
C18	0.0280 (7)	0.0218 (7)	0.0186 (6)	0.0020 (6)	0.0023 (5)	0.0028 (6)
C7	0.0202 (6)	0.0354 (9)	0.0238 (7)	0.0010 (6)	0.0074 (5)	0.0044 (6)
C5	0.0168 (6)	0.0259 (7)	0.0186 (6)	0.0004 (6)	0.0032 (5)	−0.0018 (6)
C12	0.0159 (6)	0.0311 (8)	0.0240 (7)	−0.0015 (6)	0.0010 (5)	0.0041 (6)
C11	0.0261 (7)	0.0388 (9)	0.0218 (7)	−0.0080 (7)	0.0062 (5)	−0.0035 (7)
C19	0.0343 (8)	0.0257 (8)	0.0199 (7)	−0.0005 (7)	−0.0020 (6)	−0.0007 (6)
C8	0.0219 (7)	0.0432 (10)	0.0364 (8)	0.0026 (7)	0.0077 (6)	0.0139 (8)
C21	0.0214 (6)	0.0229 (8)	0.0257 (7)	−0.0005 (6)	−0.0001 (5)	0.0025 (6)
C6	0.0158 (5)	0.0306 (8)	0.0200 (6)	−0.0047 (6)	0.0047 (5)	0.0015 (6)
C13	0.0218 (6)	0.0188 (7)	0.0263 (7)	0.0031 (6)	0.0021 (5)	0.0015 (6)
C14	0.0268 (7)	0.0207 (7)	0.0309 (7)	−0.0014 (6)	0.0035 (6)	0.0017 (6)
C9	0.0233 (7)	0.0620 (13)	0.0265 (8)	−0.0078 (8)	−0.0011 (6)	0.0178 (8)
C4	0.0169 (6)	0.0231 (7)	0.0193 (6)	0.0008 (5)	0.0023 (5)	−0.0014 (5)
C23	0.0330 (8)	0.0385 (9)	0.0166 (6)	0.0029 (7)	0.0054 (6)	0.0001 (7)
C15	0.0308 (8)	0.0258 (8)	0.0278 (7)	0.0002 (7)	−0.0028 (6)	0.0071 (6)
C20	0.0257 (7)	0.0264 (8)	0.0270 (7)	−0.0034 (6)	−0.0065 (6)	−0.0002 (6)
C25	0.0400 (9)	0.0463 (11)	0.0280 (8)	0.0159 (9)	0.0122 (7)	0.0030 (8)
C10	0.0323 (8)	0.0563 (12)	0.0194 (7)	−0.0160 (8)	0.0025 (6)	0.0005 (8)
C24	0.0395 (9)	0.0462 (12)	0.0296 (8)	−0.0040 (9)	0.0101 (7)	0.0074 (8)
C26	0.0501 (10)	0.0676 (15)	0.0227 (8)	0.0003 (11)	0.0100 (7)	−0.0118 (9)

### Geometric parameters (Å, °)

**Table:**

O1—C2	1.4277 (17)	C8—H8	0.95
O1—C5	1.4336 (16)	C21—C20	1.384 (2)
O22—C17	1.3626 (16)	C21—H21	0.95
O22—H22	0.85 (3)	C13—C15	1.526 (2)
N3—C2	1.4893 (18)	C13—C14	1.527 (2)
N3—C13	1.4897 (19)	C13—H13	1
N3—C4	1.4936 (16)	C14—H14A	0.98
C16—C21	1.3929 (19)	C14—H14B	0.98
C16—C17	1.4081 (19)	C14—H14C	0.98
C16—C2	1.5115 (19)	C9—C10	1.386 (3)
C17—C18	1.4144 (19)	C9—H9	0.95
C2—H2	1	C4—H4	1
C18—C19	1.397 (2)	C23—C26	1.534 (2)
C18—C23	1.541 (2)	C23—C24	1.537 (3)
C7—C6	1.386 (2)	C23—C25	1.539 (2)
C7—C8	1.398 (2)	C15—H15A	0.98
C7—H7	0.95	C15—H15B	0.98
C5—C6	1.504 (2)	C15—H15C	0.98
C5—C4	1.552 (2)	C20—H20	0.95
C5—H5	1	C25—H25A	0.98
C12—C4	1.5222 (18)	C25—H25B	0.98
C12—H12A	0.98	C25—H25C	0.98
C12—H12B	0.98	C10—H10	0.95
C12—H12C	0.98	C24—H24A	0.98
C11—C10	1.392 (2)	C24—H24B	0.98
C11—C6	1.398 (2)	C24—H24C	0.98
C11—H11	0.95	C26—H26A	0.98
C19—C20	1.391 (2)	C26—H26B	0.98
C19—H19	0.95	C26—H26C	0.98
C8—C9	1.389 (3)		
			
C2—O1—C5	103.91 (11)	C15—C13—H13	108.6
C17—O22—H22	107.4 (14)	C14—C13—H13	108.6
C2—N3—C13	114.66 (10)	C13—C14—H14A	109.5
C2—N3—C4	105.98 (10)	C13—C14—H14B	109.5
C13—N3—C4	112.75 (11)	H14A—C14—H14B	109.5
C21—C16—C17	119.67 (13)	C13—C14—H14C	109.5
C21—C16—C2	117.76 (12)	H14A—C14—H14C	109.5
C17—C16—C2	122.54 (12)	H14B—C14—H14C	109.5
O22—C17—C16	119.88 (12)	C10—C9—C8	119.90 (16)
O22—C17—C18	119.37 (12)	C10—C9—H9	120
C16—C17—C18	120.74 (12)	C8—C9—H9	120
O1—C2—N3	103.98 (10)	N3—C4—C12	110.54 (11)
O1—C2—C16	109.60 (12)	N3—C4—C5	102.94 (11)
N3—C2—C16	114.54 (11)	C12—C4—C5	114.32 (13)
O1—C2—H2	109.5	N3—C4—H4	109.6
N3—C2—H2	109.5	C12—C4—H4	109.6
C16—C2—H2	109.5	C5—C4—H4	109.6
C19—C18—C17	117.29 (13)	C26—C23—C24	107.39 (15)
C19—C18—C23	121.83 (13)	C26—C23—C25	107.64 (16)
C17—C18—C23	120.84 (13)	C24—C23—C25	109.81 (15)
C6—C7—C8	120.26 (16)	C26—C23—C18	111.68 (14)
C6—C7—H7	119.9	C24—C23—C18	109.35 (14)
C8—C7—H7	119.9	C25—C23—C18	110.88 (13)
O1—C5—C6	111.15 (12)	C13—C15—H15A	109.5
O1—C5—C4	103.38 (10)	C13—C15—H15B	109.5
C6—C5—C4	114.59 (11)	H15A—C15—H15B	109.5
O1—C5—H5	109.2	C13—C15—H15C	109.5
C6—C5—H5	109.2	H15A—C15—H15C	109.5
C4—C5—H5	109.2	H15B—C15—H15C	109.5
C4—C12—H12A	109.5	C21—C20—C19	119.59 (14)
C4—C12—H12B	109.5	C21—C20—H20	120.2
H12A—C12—H12B	109.5	C19—C20—H20	120.2
C4—C12—H12C	109.5	C23—C25—H25A	109.5
H12A—C12—H12C	109.5	C23—C25—H25B	109.5
H12B—C12—H12C	109.5	H25A—C25—H25B	109.5
C10—C11—C6	120.03 (18)	C23—C25—H25C	109.5
C10—C11—H11	120	H25A—C25—H25C	109.5
C6—C11—H11	120	H25B—C25—H25C	109.5
C20—C19—C18	122.28 (14)	C9—C10—C11	120.27 (16)
C20—C19—H19	118.9	C9—C10—H10	119.9
C18—C19—H19	118.9	C11—C10—H10	119.9
C9—C8—C7	119.96 (18)	C23—C24—H24A	109.5
C9—C8—H8	120	C23—C24—H24B	109.5
C7—C8—H8	120	H24A—C24—H24B	109.5
C20—C21—C16	120.36 (14)	C23—C24—H24C	109.5
C20—C21—H21	119.8	H24A—C24—H24C	109.5
C16—C21—H21	119.8	H24B—C24—H24C	109.5
C7—C6—C11	119.57 (15)	C23—C26—H26A	109.5
C7—C6—C5	122.10 (13)	C23—C26—H26B	109.5
C11—C6—C5	118.23 (15)	H26A—C26—H26B	109.5
N3—C13—C15	111.98 (12)	C23—C26—H26C	109.5
N3—C13—C14	109.53 (12)	H26A—C26—H26C	109.5
C15—C13—C14	109.52 (12)	H26B—C26—H26C	109.5
N3—C13—H13	108.6		
			
C21—C16—C17—O22	−179.25 (14)	C10—C11—C6—C5	−175.15 (14)
C2—C16—C17—O22	3.2 (2)	O1—C5—C6—C7	19.92 (18)
C21—C16—C17—C18	2.2 (2)	C4—C5—C6—C7	−96.81 (16)
C2—C16—C17—C18	−175.41 (13)	O1—C5—C6—C11	−163.77 (12)
C5—O1—C2—N3	44.04 (12)	C4—C5—C6—C11	79.50 (16)
C5—O1—C2—C16	166.94 (10)	C2—N3—C13—C15	−51.80 (16)
C13—N3—C2—O1	−152.55 (11)	C4—N3—C13—C15	−173.20 (11)
C4—N3—C2—O1	−27.52 (13)	C2—N3—C13—C14	−173.49 (11)
C13—N3—C2—C16	87.86 (14)	C4—N3—C13—C14	65.11 (14)
C4—N3—C2—C16	−147.11 (12)	C7—C8—C9—C10	0.7 (2)
C21—C16—C2—O1	91.78 (15)	C2—N3—C4—C12	124.33 (13)
C17—C16—C2—O1	−90.58 (15)	C13—N3—C4—C12	−109.47 (14)
C21—C16—C2—N3	−151.83 (13)	C2—N3—C4—C5	1.83 (13)
C17—C16—C2—N3	25.82 (18)	C13—N3—C4—C5	128.03 (12)
O22—C17—C18—C19	178.79 (14)	O1—C5—C4—N3	24.25 (13)
C16—C17—C18—C19	−2.6 (2)	C6—C5—C4—N3	145.35 (12)
O22—C17—C18—C23	−3.5 (2)	O1—C5—C4—C12	−95.68 (13)
C16—C17—C18—C23	175.10 (14)	C6—C5—C4—C12	25.43 (17)
C2—O1—C5—C6	−165.84 (10)	C19—C18—C23—C26	−0.2 (2)
C2—O1—C5—C4	−42.44 (12)	C17—C18—C23—C26	−177.83 (16)
C17—C18—C19—C20	0.8 (2)	C19—C18—C23—C24	118.53 (17)
C23—C18—C19—C20	−176.94 (16)	C17—C18—C23—C24	−59.10 (18)
C6—C7—C8—C9	0.1 (2)	C19—C18—C23—C25	−120.25 (17)
C17—C16—C21—C20	0.2 (2)	C17—C18—C23—C25	62.1 (2)
C2—C16—C21—C20	177.93 (14)	C16—C21—C20—C19	−2.1 (2)
C8—C7—C6—C11	−1.0 (2)	C18—C19—C20—C21	1.6 (3)
C8—C7—C6—C5	175.25 (13)	C8—C9—C10—C11	−0.4 (2)
C10—C11—C6—C7	1.3 (2)	C6—C11—C10—C9	−0.5 (2)

### Hydrogen-bond geometry (Å, °)

**Table:**

*D*—H···*A*	*D*—H	H···*A*	*D*···*A*	*D*—H···*A*
O22—H22···N3	0.85 (3)	1.84 (2)	2.6280 (16)	154 (2)
